# Leveraging affordances in an ecological stance: Reflective language teaching for professional development during COVID-19

**DOI:** 10.1016/j.heliyon.2023.e15981

**Published:** 2023-05-01

**Authors:** Mengtian Chen

**Affiliations:** Language and Culture Center, Duke Kunshan University, No. 8 Duke Avenue, Kunshan, Jiangsu, 215316, China

**Keywords:** Teacher professional development, Distance education and online learning, Improving classroom teaching, Teaching/learning strategies, Post-secondary education

## Abstract

The COVID-19 pandemic has made a prominent impact of social contexts on teachers' professional development in remote classroom teaching. To explore how the change has altered human-environment relationships in university language classes, this qualitative case study investigated three teachers' progressive reflection on their use of affordances for teaching Chinese as a second language (L2) during COVID-19. Under the framework of human ecological language pedagogy, three themes of emergency remote teaching emerged from monthly semi-structured interviews about the three teachers’ reflective practice in remote classrooms: computer-dominant teaching conditions, flexible classroom interaction, and rational social empathy in L2 education. The findings suggest the importance of a growth mindset for L2 teachers to leverage their teaching abilities and environmental resources for continuing professional development during COVID-19 and post-pandemic periods.

## Highlights

• Multiple-case study on the progress of remote language teaching during COVID-19.
• Adaptive teacher agency in the reflective use of pedagogical and social affordances.
• Reflective teaching as a means of self-directed continuing professional development.
• Closer interconnection between classroom dynamics and social evolution.
• Ecological language pedagogy for transformative digitalization in global education.

## 1 Introduction

The ecology of teaching and learning has been a focus of second language (L2) distance education to address the relationships between teachers, students, and the environment in remote classrooms [[1], [2], [3]], social media [[4], [5], [6]], and virtual realities [[7], [8], [9]]. Research findings highlight the importance of affordances, that is, educational opportunities arising from human-environmental interactions, to classroom pedagogies and language ideologies in social worlds [10,11]. Despite the increased social impact on L2 classroom learning [12], researchers care more about linguistic and cultural literacy [13] students are expected to acquire inside the classroom [14,15]. Teachers also find it difficult to link their classroom instruction to the society [16] to cultivate students’ L2 social capacities such as cosmopolitan dispositions in a global world [17].

The outbreak of COVID-19 in 2020 dramatically changed the ecology of L2 classroom teaching and the corresponding professional development for teachers by connecting classroom instruction more closely with social evolution [18,19]. Instead of implementing prepared teaching plans, teachers directed themselves to acquire instructional technologies while adapting face-to-face classes to remote learning platforms in a pandemic world [20]. This in turn altered the climate of teachers' professional development from standard-based, propositional achievements to self-regulated, procedural inquiries as a career goal [21]. However, a majority of teachers had insufficient knowledge and skills to utilize these novel affordances for their classroom teaching and professional development [22,23]. The literature also offered few precedents of adjusting teachers’ classroom instruction and professional trajectories along with the evolvement of a pandemic society [24].

Therefore, this study adopted an exploratory approach by tracking the remote instruction of three Chinese language teachers over 2020 to understand how they utilized the affordances for L2 remote teaching as a way of advancing their professional development during COVID-19. Levine's human ecological language pedagogy [25] was the conceptual framework of affordance analysis to examine the interplay between teachers, students, and their surroundings within the scopes of classroom, school, and the society. The ecology of L2 remote teaching was probed through small-scale, in-depth case inquiries into the dynamics of teacher agency, digital technologies, and social environment alongside the progress of remote classes, which helped depict pathways for the three teachers' professional development as the pandemic unfolded.

## 2 Background

### 2.1 Conceptual framework: affordances of L2 ecological pedagogy

Affordance is one of the core constructs in language pedagogy [26]. It is originated from the field of visual perception to denote the likelihood of an action based on one's perception of available resources rather than mechanistic reaction to the environment [27]; for example, a pool is used to drink or shower according to one's judgment of its water quality [28]. Accordingly, affordances for L2 teaching are action possibilities emerging from human-environmental interactions with the help of target language; for instance, a chair and the teacher's inviting gesture prompt students to associate a phrase with a sitting-down action in an L2 class [29]. The behavioral potentials are not created by humans nor belong to objects in the environment but reside in human perception and the ensuing actions [30]. Thus, L2 pedagogy is not just about the cognitive processing of language input and output in one's mind but social exchanges with others in particular contexts.

An ecological view of affordances in L2 pedagogy sees both symbolic and non-symbolic interactions, for instance, vocal messaging and eye contact in video conferencing, as prospective episodes for L2 communication [31]. It values more than present contextual activities that sociocultural theory proposes, such as role-plays of real-life conversations in the classroom [32] to facilitate individual development of language and literacy [33,34]. Instead, it attributes L2 achievements to the interplay between humans as agents, language as means of socialization, and multiple timescales of backgrounds as situational contexts [35,36]. Levine develops these principles into a conceptual framework of human ecological language pedagogy [25], which suggests applying affordances to L2 teaching in an ecological stance by linking classroom dynamics with that of social contexts.

Levine defines L2 pedagogy as theoretical and practical principles for a human-environmental ecology [25]: teachers and students who have distinct educational beliefs and strategies are associated with each other through different channels of co-adaptation in language socialization [37]. The notion of individual diversity aligns with the nonlinear processes of L2 learning, which makes it easier for teachers to understand what happens in the classroom and adjust their instruction accordingly [38]. The assumption of systematic variability enables teachers to respond beyond the classroom to social realities of L2 teaching, resulting in closer interconnection between school education and the society [39]. For example, global migration in the 21st century brings up issues of multilingual identities and culture adaptation that teachers need to consider when interpreting code-switching behaviors of L2 learners in class [40].

The individual and systematic complexities of human-environment relationships form the overarching sphere of Levine's framework by conceptualizing teacher capabilities, creativity, and conflict transformation and compassion in an ecological approach to language pedagogy [25]. Capabilities are more than competence, which imply teachers' personal abilities and spatiotemporal repertoire to choose what they appreciate in life rather than just their teaching aptitude and skills [41,42]. Creativity encompasses two attributes: (a) novel and original products, and (b) adaptive and collaborative processes [43], signifying the inherent creativity of language and its context-appropriate use [44,45]. Conflict transformation and compassion emphasize actionable social justice in school education [46]. Besides showing empathy for others, teachers motivate students to verbalize conflicts and come up with possible solutions [47,48]. Despite not being immediately observable in social changes, such transformative practice encourages students to discuss real-life problems from different perspectives to improve L2 social wisdom in a phenomenological manner [49].

Levine's framework provides a conceptual structure to investigate the affordances of L2 ecological pedagogy from both macro and micro perspectives [25]. Nevertheless, empirical studies to date tend to examine affordances within a closed, micro human-environmental ecology such as a face-to-face class [[50], [51], [52]] or a virtual learning platform [[53], [54], [55]]. Results are also more about teachers' discrete usage of linguistic and sociocultural affordances [[56], [57], [58]] than their pragmatic perception of integrating those affordances for classroom pedagogies [59,60]. Teacher perception is indispensable to realizing affordances in ecological pedagogy, as affordances are not available until they are noticed and attended to Ref. [61]. Perception can also be affordance itself, which is activated by reflection on the perception of human behaviors [62]. The latter meta-cognition is particularly important in distance education, where personal autonomy lays the foundation of remote community affiliation for better teaching and learning outcomes [63].

Emergency remote teaching during COVID-19 has markedly changed the affordances for L2 classroom instruction by highlighting three characteristics of ecological pedagogy in practice: (1) situational contexts, (2) teacher agency, and (3) dynamic processes [64]. Specifically, habitual face-to-face instruction was abruptly transformed online with less developed technologies and designs for exclusive remote classes after the COVID-19 outbreak [65,66]. Local realities of the pandemic continuously imposed technical and social problems on pedagogical decisions, for instance, which digital technologies may survive with the surge of Internet users, and how to manage remote classrooms under crisis circumstances [67,68]. The novel complex ecology impelled teachers to reconsider their agency [69,70], social and emotional well-beings [71,72], and teaching-learning dynamics [73] in school education. These affordance changes necessitate a re-examination of day-to-day classroom instruction to keep pace with the ever-changing educational contexts during COVID-19 [74,75], which consequently fosters the research and practice on teachers’ professional development from a continuing, realistic perspective [76].

### 2.2 Professional development by means of reflective teaching

The novel affordances brought by COVID-19 facilitate L2 teachers to direct themselves through remote teaching as a way of continuing their professional development in a pandemic world. Continuing professional development (CPD) is a type of professional education that teachers receive after entering the workforce to enhance teaching skills and visions as part of their career goals [77], for example, digital networking for educational reforms in modern society [78]. Self-directed CPD makes the most of teacher agency [79] with either sole or collective spontaneous professional learning activities, for instance, reading summaries of pedagogical research on one's own and sharing experience within teacher communities [80]. Apart from attending sit-and-get workshops that deliver knowledge to a broad audience, teachers continually reflect on their classroom instruction to customize what they learned from those workshops to their own teaching [81]. Thus, teachers who carry out self-direct CPD do not passively receive general instructions from a top-down authority but actively construct on-demand knowledge and skills with respect to realistic instructional contexts in a bottom-up manner.

The transformative attribute of self-directed CPD calls for teachers to incorporate professional learning into daily instruction via approaches such as reflective teaching [82]. Reflective teaching, a procedural approach to learning by doing [83], aligns with ecological language pedagogy: teachers proactively actualize while gaining insights from reflection on daily instruction to engage students in critical thinking of linguistic creativity and sociocultural literacy [50,84]. Furthermore, reflective teaching extends here-and-now affordances [85] to include retrospective experience and future anticipation of human-environmental ecology in education [86]. By situating affordances on a continuous timeline, teachers are more likely to put themselves in students’ shoes and embrace the ethnographic and anthropological properties of L2 pedagogy, that is, language teaching with regard to the historicity and subjectivity of sociocultural forces shaping personal ideologies and community affiliations in society [31].

Reflective teaching has been conceptualized into different frameworks according to its temporal [87,88] and relational features [89,90]. A few models, such as Gibbs's reflective learning cycle [83], have been transformed into guidelines for L2 remote teaching during COVID-19 [91,92]. Nevertheless, most reflective practice targeted student learning rather than teachers' professional growth, for instance, think-aloud learning for linguistic literacy [93] and structured journaling for the development of L2 selves [94]. Among the limited number of teacher studies, collective traits of teacher agency, such as self-regulation and leadership, were more discussed than actions of individual teachers [95,96]. Few of them addressed the sociocultural influence of classroom teaching as well, which is, however, one of the critical elements of ecological pedagogy [97,98].

Implementing L2 ecological pedagogy through reflective teaching encourages teachers to pursue CPD in aspects such as innovation in the classroom [99,100], identity commitment [101,102], and social and cultural wellness [103,104]. Teacher agency was the focus of inquiries under frameworks such as activity system and sociocultural theory [105,106]. A majority of studies examined teachers’ articulated agency in retrospective interviews [95,107,108], whereas actual alternation of agency in response to affordance changes in the classroom were less investigated [109,110]. Given the shared emergent features of L2 ecological pedagogy and self-directed CPD, it is necessary for teachers to conduct reflective teaching with beliefs about sustainable professional development while making a difference to the ecology of teaching within and beyond the classroom.

### 2.3 Current study

In view of the foregoing, this study tracked the remote teaching of three Chinese language teachers during COVID-19 to examine how they carried out self-directed CPD with the affordances of L2 ecological pedagogy in an exploratory virtual environment. Affordances were defined as actionable opportunities for classroom instruction and professional development, which teachers identified from both course delivery and social evolution during the pandemic. The reasons for investigating Chinese language teachers in mainland China were: (1) as a less commonly taught L2, Chinese has been less investigated than European languages regarding digital affordances for classroom teaching [111]; (2) mainland China was one of the first places where higher education was severely affected by the COVID-19 outbreak in 2020. Hence, teachers’ handling of the novel affordances in this area may enlighten L2 remote teaching in other regions of the world.

The study addressed the following two research questions:

1. What pedagogical and social affordances did teachers use to navigate themselves through remote teaching as a way of advancing self-directed CPD during COVID-19?
2. How did they link the dynamics of L2 remote classes to the ever-changing social contexts in a pandemic world?

The implications of investigating teachers' self-directed CPD in L2 remote teaching during COVID-19 are two-fold. Theoretically, it substantiates the application of L2 ecological pedagogy at different levels of a complex social system, which in turn grounds Levine's framework [25] on the ecological properties of affordances, such as the integrated use of teacher capabilities, creativity, and social compassion in classroom teaching [112]. Practically, it provides examples of how to conduct L2 remote teaching in an ever-changing social environment while advancing teachers' CPD through self-reflective daily instruction rather than authoritative extracurricular trainings [24]. These teaching experiences could not only guide teachers to manage their remote classes during COVID-19 [113], but also foster their awareness of an affordance-rich classroom and its social correlates [51]. The latter affordance is essential to building rapport among teachers and students, thus leading to more community-based, resilient teaching against educational uncertainties in the future [114].

## 3 Method

### 3.1 Context

Owing to the outbreak of COVID-19 in January 2020, all the universities in mainland China were notified to move online two weeks before the beginning of spring semester [115]. This resulted in a scramble to remote teaching by L2 teachers whose students were scattered in different time zones and whose classroom instruction relied heavily on face-to-face interaction. Digital technologies were less developed for exclusive remote language classes upon the COVID-19 outbreak, such that teachers had to explore new platforms and techniques as the pandemic went on [116]. Although most college teachers had taught online courses before [117], they still demanded technical and pedagogical instructions to streamline their remote teaching and professional development during COVID-19 [118,119].

### 3.2 Participants

This study was approved by Duke Kunshan University Institutional Review Board for ethical considerations of educational research involving human subjects. Considering short preparation time, difficulties of collecting longitudinal data during COVID-19, and the exploratory nature of the study, the researcher combined purposive sampling with convenience sampling to recruit a small number of participants for in-depth case inquiries [120]. A candidate pool was first created by searching through the researcher's academic network according to two criteria: (1) teachers in their early 30s with less than three years of work experience, as young novice teachers are more comfortable with the Internet and would be more engaged in remote teaching [16]; (2) teachers who would teach Chinese language courses or linguistic courses to non-native speakers in spring. Then the researcher reached out to individual candidates for their confirmation of participation. Meanwhile, maximum variation sampling [121] was carried out by selecting participants who taught distinct levels of courses from different universities to ensure the richness of L2 remote teaching data in a broad sense.

Finally, three female teachers from three local Chinese universities participated in the study. Teacher Z (pseudonym, the same below) taught Second-Year Chinese. Teacher S taught Fourth-Year Chinese and another course to student teachers in teaching Chinese as an L2. Teacher H taught two linguistic courses, namely Modern Chinese and Bilingualism. All three participants had their remote classes twice a week, with each class lasting 80–90 min in a 17-week spring semester. Teacher Z had eight non-native students in each of her three class sections. Teacher S had 15 non-native students in Fourth-Year Chinese but 30 native and non-native student teachers in the other course. Teacher H had a mixture of native and non-native students in her two courses: 50 in Modern Chinese and 20 in Bilingualism.

### 3.3 Design

Multiple-case study methodologies were adopted with each participant as the unit of analysis [122,123] to examine reflective teaching processes of each case while discovering patterned affordances of L2 ecological pedagogy for teachers' self-directed CPD during COVID-19. Semi-structured interview was employed as the primary data collection method because: (1) it could elicit thick descriptions of contextual behaviors with certain framework parameters [124]; (2) it could facilitate participants to recount lived experiences without feeling confined to interview structures [125]; and (3) it could promote reciprocal exchanges between the researcher and participants, by which participants could provide additional contexts to the researcher's data interpretation while the researcher's questions may enlighten participants to reflect on their actions further [126]. Furthermore, participants' self-reflective notes and videos of classroom teaching were recorded to triangulate interview data for more trustworthiness of the study [127].

Interview topics were designed based on the four components (i.e. contextual complexity, capabilities, creativity, and conflict transformation and compassion) of human ecological language pedagogy [25] to capture participants' agentic use of pedagogical and social affordances for L2 remote teaching during COVID-19. Questions in each interview were arranged according to the progressive stages of Gibbs's reflective cycle [83] (see Appendix A for a complete list of interview questions), for instance, “how was your first-month remote teaching” for description, “what accommodations have you made to respond to those issues” for analysis, and “what do you expect for the remote classes of next month” for action plans. The sequence of interviews for each participant formed the same cycle from the exploration of emergency remote teaching to its adaptation and anticipation for the future.

### 3.4 Procedures

Each participant signed an electronic letter of informed consent before the beginning of the study in early February 2020. They were asked to provide pseudonyms of their names and institutions for the researcher to present their reflective teaching data to the public without disclosing their identities. Permissions were sought in advance if additional non-verbal information not elicited from interviews, self-reflective notes, and classroom videos, such as pictures used in classroom activities, was needed for data analysis [128].

Each participant had a 30-min interview in each month of the study, resulting in four interviews in spring and another follow-up interview in fall. The total length of data collection period was eight months. Interview schedules followed the progress of remote teaching during COVID-19: conversion to online classes, one-month adaptation to remote instruction, Internet fatigue at mid-term, and back to campus. During the interviews in spring, participants first answered each question based on their use of affordances for remote classroom instruction, which was probed further if any points of self-directed CPD were raised in their answers. Questions in the follow-up interview prompted participants to revisit their experiences of emergency remote teaching and its impact on their CPD in the long term. Participants also kept self-reflective notes and videos of their classroom instruction in spring, which were saught afterwards to clarify or complement their interview answers.

Interviews were conducted and recorded remotely on WeChat, a Chinese social media app, to align with self-quarantine policies in mainland China during COVID-19. Since the starting date of spring semester was the same (February 10) in each participant's university, their interview dates in spring were almost the same to represent similar stages of emergency remote teaching. The date of the follow-up interview was selected by each participant at the end of the first month of fall semester in September 2020. Self-reflective notes and classroom videos were delivered via email upon request in the later stage of data analysis.

### 3.5 Analysis

After all the interviews were finished, the researcher transcribed verbatim the recordings in Chinese, the dominant language used for interviews. Thematic analysis was then carried out through three steps of coding, sorting, and synthesizing affordance data [129]: first, the researcher labeled participants' answers according to the arrangement of interview questions, namely Gibbs's reflective cycle for classroom instruction [83]. Each portion addressing research questions was extracted along with a note to explain its relation to participants' self-directed CPD. Next, the researcher went through an iterative process of reading, comparing, and summarizing those excerpts, from which patterns of affordance use emerged. Finally, the researcher interpreted these affordance data with respect to participants' self-directed CPD to come up with three themes of reflective remote teaching under the framework of human ecological language pedagogy [25].

Besides data triangulation, a few other methods were employed to guarantee the trustworthiness of the study [130]. The format and content of questions for each participant in each interview were similar, which was equivalent to collecting multiple points of data from different subjects for within-case and cross-case comparisons. Before the initial coding, transcripts were emailed to each participant to check the content for data credibility. All three participants responded within 14 days by adding words that the researcher sought for clarification. In addition to the researcher, another Chinese language teacher who did not participate in the study was asked to carry out the same analysis procedures independently after being informed of research purposes to ensure dependability in qualitative data analysis. The researcher and the teacher met afterwards to discuss and resolve discrepancies in coding, refine data interpretations, and arrive at an agreement for emergent themes in the end.

## 4 Results

Three affordance themes, six sub-themes, and 12 codes were extracted from thematic analysis of interviews, self-reflective notes, and classroom videos as shown in Table 1. The three themes, namely computer-dominant teaching conditions, flexible classroom interaction, and rational social empathy, corresponded to the four components of human ecological language pedagogy [25]: contextual complexity, human capabilities and creativity, and social compassion. Affordances of capabilities and creativity were categorized into one theme (i.e., flexible classroom interaction) to demonstrate participants’ use of teacher agency in remote classroom instruction. The two sub-categories of each theme contained answers to two research questions respectively, that is, the pedagogical and social affordances participants identified and utilized, and the efforts they made to link classroom teaching to the society in light of self-directed CPD during COVID-19. Quoted interview excerpts below were translated into English by the researcher (see Appendix B for original transcripts in Chinese).

**Table 1:** Themes and codes denoting participants’ reflective remote teaching during COVID-19.

Theme	Sub-theme	Code
Computer-dominant teaching conditions	Technology use in remote schooling	Digital learning platforms
		Self-study opportunities
	Work-life balance	Social media apps for schooling
		Data security of remote classes
Flexible classroom interaction	Synchronous online classes	Classroom activities
		Class participation rules
	Asynchronous self-regulated teaching and learning	Progressive feedback
		Remote assessment
Rational social empathy	Classroom discussion on social issues	Multi-literacy in Chinese
		Affective bonds in L2
	Experiential teaching in virtual contexts	Technical aptitude
		Adaptive mindset

### 4.1 Computer-dominant teaching conditions

One of the major affordance changes from face-to-face to emergency remote teaching was that the Internet was converted from a supplementary tool to the primary medium of classroom instruction. Consequently, course design and classroom management needed to be restructured in the virtual environment. Classroom learning was also stipulated to be optional by school, given the severity of the educational disruption, so that teachers could not require students to attend classes and submit assignments as usual. While these policies helped reduce the school anxiety of students, they actually put teachers in a dilemma: eager to support students’ remote learning but concerned about pushing them too much.

To tackle the complexity of computer-dominant teaching conditions during COVID-19, participants adjusted their classroom management to reconcile the demand for teaching effectiveness with constraints of remote classes. Teacher Z gave an example of Second-Year Chinese in her third interview (“R” stands for the researcher, the same below):

Excerpt 1: Backup plans for online class attendance.

Z: We have extra credit, like, they record videos or write an essay.

R: This is what was required in the syllabus at first, isn't it?

Z: No, it came up last week.

R: Why you, why did you suddenly come up with this?

Z: Our teaching cohort decided it. Students were absent from online classes sometimes due to the pandemic situations in their countries. Last week, the university wanted them to report how they were doing with remote courses and self-quarantine at home. Then we thought we could ask our students who missed the classes to describe daily routines in Chinese or to videotape self-quarantine stories with Chinese captions to count as making up lessons. It is also a good opportunity for them to use L2 in real life.

Besides classroom pedagogies, remote instruction also complicated socio-educational issues such as work-life balance for teachers, which Teacher S explained in her first interview:

Excerpt 2: Overuse of non-academic digital tools for remote schooling.

S: I don't like to use WeChat for work.

R: But this is ubiquitous in local Chinese universities.

S: Right, but the boundary between work and life becomes blurred. Even though I have blocked students in my moments and channels, I can still receive messages from my class groups at about 2:00 am.

R: Well, you can mute group notifications and turn off your phone when you sleep.

S: Yeah, but how about the daytime when you are off work? How do you feel when you are eating out with friends and suddenly, a WeChat message pops up that there are something wrong with your students? It is more common for my L2 classes due to the time difference.

The overuse of a single digital tool for both academic and social communication raised the risk of technical breakdown when tons of users were active at once. To cope with this, technology companies, according to Teacher S's and Teacher H's self-reflective notes, developed a way of joining in online classes without login information by scanning a quick response (QR) code or clicking a link that was open to everyone. Despite being more convenient, this technique stirred up worries about the data security of online classes, as Teacher H complained in her second interview:

Excerpt 3: Data security of online classes.

H: In online classes, all you said will be recorded. The mental burden is heavy. If you accidently say something inappropriate, it will be very uncomfortable.

R: But you can always correct it afterwards.

H: Yeah, but I am scared of these remarks to be disseminated elsewhere. I dare not to talk about something, either. There is a course that requires students to read English articles. Once they asked me where to find those articles. I said Google Scholar. Then they asked whether they should use virtual private networks (VPNs) to access Google, from where I dared not to continue further. VPN is officially forbidden in mainland China. I am just back. I do not know how far I can go with this topic.

### 4.2 Flexible classroom interaction

A subsequent change of pedagogical affordances brought by alternation of instructional settings was the decline of synchronous classroom interaction. This may be due to the difficulty of exchanging information remotely, but having students study at home rendered them more likely to lay back instead of reaching out to communicate. To maintain teaching efficiency in remote contexts, participants transformed classroom activities according to course content and student levels: handwriting exercises in Teacher Z's and Teacher S's L2 classes were cut down because of the inefficiency of coordinating them online. Conversely, experimental praxes in Teacher H's linguistics courses were enhanced as they were more convenient to be operated with multimedia; for example, students enrolled in Bilingualism were asked to restore the procedures for a vocabulary learning experiment with E-prime, a software for reaction time tests.

Despite different operations of synchronous classroom interaction, participants’ rationale for lecture-practice balance in remote teaching was similar. A flipped design was implemented in L2 classes to compensate for reduced learning opportunities caused by the decrease of synchronous classroom exercises: teachers saved the time of vocabulary and grammar lecturing for drills and task activities while students were required to study words and patterns by themselves before class. As for linguistics classes, Teacher H introduced more experimental exercises to motivate student learning, which at the same time took up time for faster lecturing due to diminished opportunities for teacher-student interaction in class.

**Fig. 1:**
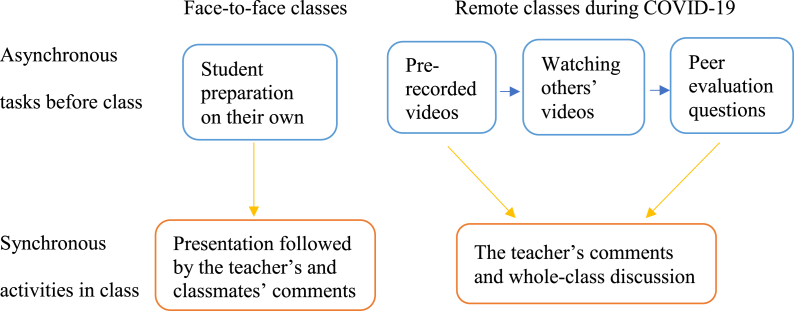
Oral performance procedures in face-to-face classes and remote classes during COVID-19.

Another change of social affordances was the increased importance of personal autonomy: teachers and students needed to manage schoolwork while adapting to online and offline environments by themselves during COVID-19. To facilitate self-regulated teaching and learning at home, participants created a variety of asynchronous opportunities for classroom interaction, which was observed from Teacher Z's classroom videos and described as shown in Fig. 1 in her third interview:

Nevertheless, both students and teachers may be overwhelmed by double workload when incorporating asynchronous learning steps and progressive feedback into classroom activities, as participants wrote in their self-reflective notes, since what could have been delivered multi-dimensionally in person was all documented digitally for remote information exchange.

In addition to intellectual scaffoldings, participants also reformulated classroom management rules to appreciate class participation more than attendance, which Teacher H exemplified in her second interview:

Excerpt 4: Participation rules in online linguistics classes.

H: Participation takes up 10% of final grade. Scores in each class session are divided into three levels, namely 0, 7, and 10. Zero means you stay silent for the whole class. As long as you voluntarily speak up, you will get 7 points. If your comments are really thoughtful, you will get 10 points. The final participation score is not averaged across class sessions, but the highest score you have ever got. Most of my students are accustomed to exam-oriented education since childhood, so they are stimulated to “swipe” their participation scores: someone who have not expressed themselves tries hard to grab the chance to speak, and those who have already got 7 points continue to contribute more in class.

Nonetheless, participants diverged on assessment criteria for student achievements in remote classes: Teacher Z regarded scores as an indication of learning efficiency. She provided many opportunities for students to practice Chinese and elevate their scores, but she did not change her grading standards despite the emergent Internet fatigue during COVID-19. By contrast, grades were dispensable for Teacher S to evaluate her students’ remote learning. They were more meaningful for students themselves to reflect on self-study progress and modify learning strategies when necessary. To Teacher H, scores did reveal something, but other objective realities may affect student performance as well: ease of access to learning resources, familiarity with course materials, and school environment to name a few.

### 4.3 Rational social empathy

A unique affordance for emergency remote teaching was the strengthened relationships between classroom instruction and the society. However, participants were critical of incorporating COVID-19 events into course materials. Part of the reason was the risk of aggravating the inequality of Internet access and socioeconomic status among students, but more weight was put on what to teach and how to lead L2 classroom discussion on social topics.

For Second-Year Chinese, Teacher Z directed student attention to multi-literacy embodied in interpreting COVID-19 news. She introduced a few examples in her third interview:

Excerpt 5: Multi-literacy in remote Chinese language classes.

Z: Quite a few opportunities exist behind the news for students to acquire multi-literacy in Chinese during COVID-19. Even though they are in Second-Year Chinese, the Intermediate level, I still encourage them to think beyond what have been reported in news, for instance, the handwriting Chinese poems attached to donation boxes sent to Wuhan, and to discuss why it happened, its social and cultural implications, and how they would respond if they were in Wuhan. As for linguistic literacy, they (students) can practice listening skills, like recognizing the number of positive cases and picking up proper names of administrative districts in Chinese from daily news reports. To connect linguistic skills with sociocultural practice, I often lead them to analyze the wording of news in multiple contexts, like how the degree of formality varied when broadcasting to different groups of audience, and why general words instead of specific vocabularies were used when reporting confirmed cases of COVID-19 in Chinese.

Another unique social affordance was the reinforced affective bonds between teachers and students in L2, which, however, may interfere with classroom instruction if being handled inappropriately. To optimize its positive impact, Teacher S guided students to express rational compassion for social issues, which she explicated in her fourth interview:

Excerpt 6: Transformative classroom practice with COVID-19 events.

S: I led a 10–15 min discussion at the beginning of each week on the pros and cons of current COVID-19 prevention measures in different countries. Students really liked it.

R: To keep pace with the ever-changing social environment, ah?

S: Yes. I know they want to show empathy for the suffering people while venting their own emotions, but they can go a step further to apply what they learned in class to tackle realistic problems.

R: True, these are very good authentic materials for critical thinking.

S: Not just about that, but using L2 to express sentiments in topics such as ideologies and social justice, or for student teachers in my language teaching course, to develop backup plans if another global health crisis like COVID-19 takes place in the future. Considering the current pandemic situations, we need to think beyond the classroom to broaden our minds and keep up with the evolution of modern society.

These affective experiences were less attainable in normal classes due to the difficulty of bonding L2 with sentiments [131]. However, they had the potentials to activate another cognitive channel for L2 learning by empowering students to explore real-life situations at an emotional distance while developing morals worthy of L2 knowledge and skills.

Given the theoretical orientation of linguistics courses, Teacher H did not touch much upon the links between course materials and social issues during COVID-19. Nonetheless, she developed her abilities to handle unexpected classroom incidents on the Internet, which was also cultivated from utilizing social affordances for emergency remote teaching. She reflected on this in her follow-up interview:

Excerpt 7: Adaptation to unexpected incidents in remote classes.

H: I can be more tolerant of some incidents now, like not easily getting frustrated with anything unusual. If students found the Internet was slow, I would say no worries that you could watch the playback of class videos afterwards. Sometimes something was wrong with my microphone, and then students would calmly remind me that they could not hear me. Or sometimes it was just the problem of my lecturing. But anyway, I am now okay with a whole bunch of circumstances.

This professional growth resulted not just from increased familiarity with remote instructional technologies and skills, but from the development of an adaptive mindset, that is, to accept imperfection in teaching but to watch out for opportunities to improve it.

## 5 Discussion

This study investigated the reflective use of affordances, which were treated as benefits and challenges of remote classroom instruction by three Chinese language teachers during COVID-19. Pedagogical and social affordances were analyzed under the framework of human ecological language pedagogy [25] regarding the three teachers’ utilization of their capabilities, creativity, and social compassion to tackle the complexity of affordances in both classroom and social settings. Reflective teaching has proven feasible as a means of self-directed CPD that values human-environmental interactions in daily instruction [77], which can answer the research questions of the study as follows.

### 5.1 Pedagogical and social affordances for remote classroom teaching

All three teachers responded actively to the changes of affordances for classroom teaching, which was triggered by the abrupt pedagogical transition online and the rapid evolvement of social contexts after the COVID-19 outbreak. For example, Teacher Z and Teacher S flipped their L2 classes to cope with the decrease of in-person language practice by incorporating asynchronous self-paced coursework into classroom activities. This use of teacher agency as reaction to affordance changes implied the pragmatic properties of teacher capabilities rather than their functioning as intrinsic teaching competence [132]. Conversely, affordance changes invited certain instructional behaviors to be more likely to occur than others [133]; for example, emergent teaching plans replaced prefabricated course designs because of few precedents and more uncertainties in emergency remote teaching. These external forces helped reveal the mutual dependence of agency and affordance as part of teachers’ self-directed CPD in L2 ecological pedagogy, which indicated the phylogenetic nature of human-environment relationships in a complex system [112].

Nonetheless, the three teachers differed in their use of affordances as a result of distinct teacher beliefs and objective factors such as course content in remote classroom instruction: Teacher Z and Teacher S reduced handwriting exercises because of the difficulty to monitor them remotely, whereas Teacher H reinforced experimental practice with the help of multimedia in online class sessions. Meanwhile, Teacher Z and Teacher S diverged on their interpretations of student scores as they had different assessment principles for remote classes. These differences indicated the individual diversity of teacher capabilities and creativity in a synchronic sense, which has been evidenced in previous research on both normal and emergency remote teaching [134,135].

Furthermore, the study revealed teachers’ reflective use of affordances as their progressive adaptation to the contextual complexity of remote teaching during COVID-19: Teacher H gradually gained more tolerance and skills to handle unexpected classroom incidents in the virtual environment. Likewise, Teacher Z added extra credit activities as follow-ups on revised school policies at mid-term for students to make up lessons while practicing L2 in realistic situations. These adjustments implied the nonlinearity of teacher capabilities and creativity for self-directed CPD in a diachronic sense, which, together with individual differences of teacher agency, embodied the ontogenetic nature of human-environmental ecology in a complex system [136].

### 5.2 Socio-educational connections in L2 ecological pedagogy

When it comes to the links between classroom instruction and the society, the human-environmental interactions performed by the three teachers in emergency remote teaching substantiated the principles for human ecological language pedagogy at three levels [25]. At micro level, designing classroom activities based on COVID-19 events not only raised students’ awareness of social justice in coursework but also assisted them in acting on it at an emotional distance [137]. For example, Teacher S spent 10–15 min each week to lead whole-class discussions about COVID-19 prevention measures in different countries and asked students to come up with possible improvements of those measures. This kind of transformative practice, though not immediately implemented as reforms in current society, encouraged students to think beyond the classroom to apply learned L2 knowledge and skills to realistic situations.

More importantly, all three teachers regarded the complexity of social contexts as part of school education instead of outside elements that contributed to its ecology [138]. This meta-cognition of affordances facilitated them to transform classroom practice to educational endeavors at a meso, curriculum-based level [139]. For example, Teacher Z and Teacher S incorporated COVID-19 experiences into L2 classes with distinct course objectives: for Teacher Z's Second-Year Chinese, it was the acquaintance with Chinese words and patterns to describe what happened in the society. For Teacher S's Fourth-Year Chinese, they delved more into the social implications of COVID-19 as a public health crisis and its accompanying educational disruption for higher-order thinking and practice in L2. This critical pedagogy supported teachers in navigating through the pandemic while staying alert to emergent affordances for remote teaching in a socially informed manner [140].

At macro level, addressing the social affordances for emergency remote teaching promoted the cultivation of cosmopolitan citizenship in school education [17]. By exposing students to COVID-19 issues in the classroom, Teacher Z and Teacher S guided them to develop compassionate dispositions in L2 while appreciating the variety of sociocultural perspectives from a global point of view. This cultivation of educational ideologies also rendered teachers themselves to develop an adaptive mindset to handle uncertainties such as Internet fluctuation and work-life conflicts in remote classroom teaching. These joint efforts built up learning communities in virtual contexts with intellectual exchanges and affective bonds against the COVID-19 pandemic, which, despite being opaque to students and teachers at first glance, may make a difference to the transformative potentials of educational rationality for human flourishing in modern society [141].

**Fig. 2:**
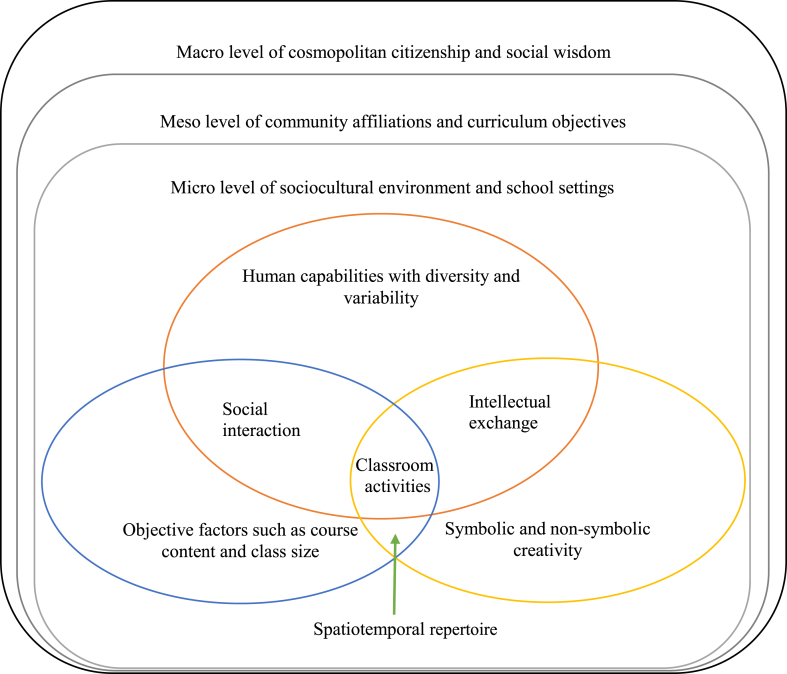
A complex system of affordances in human ecological language pedagogy.

A graphical summary of the three teachers' use of affordances for remote teaching during COVID-19 under Levine's framework of human ecological language pedagogy [25] is illustrated in Fig. 2.

### 5.3 Implications and limitations

The findings of the study contribute to the literature on L2 teachers' CPD in three aspects. Theoretically, they corroborate a context-specific, transformative conception of self-directed CPD [16,142] by demonstrating teachers’ adaptive realization of pedagogical and social affordances for remote teaching during COVID-19. This in turn substantiates the framework of human ecological language pedagogy [25] with principles for enacting teacher agency in the classroom, for instance, flexible formats of teacher-student interaction, and discussion on social issues at an emotional distance. Methodologically, reflective teaching narrows the gap between research and practice on self-directed CPD, by which teachers enhance their teaching techniques and beliefs through procedural inquiries of professional knowledge and its contextual use [143]. This sort of reflective practice is mostly robust when teachers carry out instructional observation and reflection in a world of non-linear complexity such as the COVID-19 pandemic [144].

In practice, teachers’ self-directed CPD through reflective remote teaching promotes digital transformation of higher education via a digital teaching and learning space in and out of class [145]. By fostering dual digitalization, namely digitalization of education and its stakeholders [146], teachers apply digital technologies in the classroom, extend academic and social exchanges with students beyond the classroom, and integrate school education with the society. Such multi-level alignments not only invigorate classroom instruction in terms of its technical infrastructure and pedagogies [147], but also add to the sustainable development of L2 distance education at universities, which is co-constructed by human capabilities, semiotic creativity, and sociocultural environment in a complex system of the world [148,149].

This study was an exploratory research to investigate teachers' self-directed CPD through small-scale, longitudinal case inquiries. Limitations existed as to the representativeness of sampled L2 teachers in remote teaching during COVID-19. More large-scale studies are necessary afterwards to generalize the implications of pedagogical and social insights gained from this study in mainland China to similar instructional contexts in other regions of the world. Direct classroom observation and student feedback can be collected as well to complement teachers’ self-reflective comments on their implementation of L2 ecological pedagogy.

## 6 Conclusion

This study explored the self-directed CPD of three Chinese language teachers in mainland China with respect to their reflective use of affordances to tackle computer-dominant teaching conditions, remote classroom interaction, and social contexts during COVID-19. The progressive diversity and variability of pedagogical and social accommodations initiated by the three teachers were an integrated reflection of their educational ideologies, curriculum designs, and teaching strategies as the pandemic evolved. The nonlinear human-environmental dynamics emerging from reflective teaching substantiated the conceptual framework of human ecological language pedagogy through the interplay between teacher capabilities, creativity, and social compassion in a complex system, which also provided transformative implications for teachers’ self-directed CPD in the global trend of digitalization in school education.

Future studies can work on the modeling of L2 ecological pedagogy with quantifiable cross-sectional data while dealing with its practical challenges as to maximize the availability of affordances to a larger population of teachers in various instructional settings [25]. Commonalities of optimal affordance use need to be discovered in such topics as how to ascribe teaching accommodations to student achievements and how to induce rational social empathy in remote classrooms. Orienting toward a more humanistic approach, future research may also address the multilingual identities of students and teachers as another means of leveraging affordances in an ecological stance [150].

## Author contribution statement

Mengtian Chen: Conceived and designed the experiments; Performed the experiments; Analyzed and interpreted the data; Contributed reagents, materials, analysis tools or data; Wrote the paper.

## Data availability statement

The data that has been used is confidential.

## Declaration of competing interest

The authors declare that they have no known competing financial interests or personal relationships that could have appeared to influence the work reported in this paper.
