# Supplementary material for: Leveraging affordances in an ecological stance: Reflective language teaching for professional development during COVID-19
Source: Heliyon. 2023 May 1;9(5):e15981. doi: 10.1016/j.heliyon.2023.e15981 (PMC10150415; doi:10.1016/j.heliyon.2023.e15981)
Supplement: Multimedia component 1 [file mmc1.docx]

**Appendix A: Questions in Semi-Structured Interviews**

Below is a list of open-ended questions the researcher asked each participant during the interview. The interview content of each participant were different based on their answers to these questions and their further elaborations on related topics.

Interview 1:

1. Could you tell me something about your remote course(s) this semester, the content, format, learning platforms, etc.?
2. What are the differences between your normal face-to-face classes and remote classes during COVID-19?
3. How do you adapt your face-to-face instruction to emergency remote teaching?
4. What are the issues or concerns you have now?
5. What do you expect for the 2020 spring semester?

Interview 2:

1. How was your first-month remote teaching?
2. You mentioned ___________ in your first interview. Are you still worried about it now?
3. Are there any new issues popping up in the first month of your remote teaching?
4. What accommodations have you made to respond to those issues?
5. What do you expect for the remote classes of next month?

Interview 3:

1. How was your teaching in the past month? Are there any changes of administrative arrangements in your university?
2. You mentioned ________ in your second interview. Are you still worried about it now?
3. Is there any Internet fatigue experienced by your students or yourself? How do you cope with that?
4. Are there any changes of instructional technologies and strategies applied to your remote course(s)? Why or why not?
5. What are your expectations for the remaining period of spring semester?

Interview 4:

1. Could you tell me the administrative arrangements of your university for the last month of spring semester?
2. Does your university have any plans for returning to campus? If yes, how? If no, why?
3. What is your anticipation of the transition back to campus? How is it related to remote teaching during COVID-19?
4. What are your students’ course achievements in the 2020 spring semester?
5. How do you evaluate your teaching in spring?

Follow-up interview:

1. How is your teaching in fall?
2. Have you experienced any “reversed shock” after returning to face-to-face classrooms? If yes, how long did it last? If no, why?
3. What are the differences between face-to-face classes now, those before the COVID-19 outbreak, and remote classes during COVID-19?
4. Do you think the remote teaching during COVID-19 has any backwash effects on your face-to-face instruction now? Why or why not?
5. What do you envision for your classroom instruction and professional development in the post-pandemic era?
