# Supplementary material for: Leveraging affordances in an ecological stance: Reflective language teaching for professional development during COVID-19
Source: Heliyon. 2023 May 1;9(5):e15981. doi: 10.1016/j.heliyon.2023.e15981 (PMC10150415; doi:10.1016/j.heliyon.2023.e15981)
Supplement: Multimedia component 2 [file mmc2.docx]

**Appendix B: Chinese Transcriptions of Quoted Interview Excerpts**

Teacher Z

Excerpt 1: Backup plans for online class attendance

Z: 我们有额外的加分项目，就是让他们录一段视频或者写一篇文章。

R: 这是一开始就在课程大纲里规定好的，对吧？

Z: 不是，这个是上周才跳出来的。

R: 为什么你，为什么突然想到用这个？

Z: 是我们教研组一起决定的。学生因为他们国家的疫情原因，有的时候不能来上网课嘛，然后上周学校就让他们说，来汇报一下他们都是怎么上网课、怎么居家隔离的。然后我们就想着可以让学生用中文来说一说，他们每天的日常活动，或者拍一个小视频，加点中文字幕，这个就可以算作他们补课这样。也是一个在现实生活中用二语的好机会。

Excerpt 5: Multi-literacy in remote Chinese language classes

Z: 对学生来说，在疫情期间，有很多学习中文以及它背后的社会文化内涵的机会。虽然他们只有二年级，就是中级汉语的水平，我还是会鼓励他们去思考新闻背后的一些东西，你比如像给武汉捐赠物资的箱子上面贴的中文诗词，还是用手写的，那我就会让他们讨论一下，为什么要这么做，它可能带来的社会和文化影响是什么，如果你当时在武汉，你会怎么回应。至于语言上的学习，那他们就可以通过每天看新闻来练习听力，你看像听每天的确诊人数来听辨数字，还有学习一些中文里专有的世界行政区域名词。如果要把语言和社会文化学习结合起来，我就经常带着他们分析各种语境下的新闻报道，你看像它用词的正式程度，是怎么随着观众群体的变化而变化的，还有为什么在用中文播报确诊病例的时候，要用笼统的词汇，而不是非常具体的那种。

Teacher S

Excerpt 2: Overuse of non-academic digital tools for remote schooling

S: 我挺不喜欢用微信来工作的。

R: 但这个在国内高校很普遍啊。

S: 对，但是这个工作和生活的界限就很模糊。即使我把学生屏蔽了，他们看不到我的朋友圈和频道，我还是会在，就是凌晨两点收到班级群里的信息。

R: 那，其实你可以把群消息屏蔽了，然后在睡觉的时候关机就好了。

S: 对，但是你白天下班的时候呢？比方说你和朋友出去约饭，然后你突然收到一条微信说，你的学生出问题了，那你该怎么办？我的留学生班这种情况更多，因为有时差嘛。

Excerpt 6: Transformative classroom practices with COVID-19 events

S: 我每周一开始会抽10到15分钟，来和学生讨论一下，现在各个国家的一些防疫政策的优劣。学生还挺喜欢的。

R: 就是关注社会时事呗？

S: 对。我知道他们想和那帮，就是疫情比较严重的国家的人共情嘛，然后也借机发泄一下他们的情绪啊什么的，不过我是想，他们还可以更进一步，就是用他们在课上学到的语言啊知识啊什么的，来探讨实际问题。

R: 对，这些都是很好的训练批判性思维的真实材料。

S: 不光是这样，就还有是用他们的第二语言，来讨论一些意识形态啊社会公平正义之类的更深层次的话题，或者对我的语言教学课上的本科生来说，可以想一想，如果将来又有一个像新冠这样的全球性的危机出现的话，他们可以有什么应对策略。就当前的疫情情况来讲，我们不仅要思考这个课怎么上，还得想一想更大方面的事情，与时俱进嘛，对吧。

Teacher H

Excerpt 3: Data security of online classes

H: 网课的话，你说的话都会被录下来，所以心理负担就很重。如果你不小心说了什么不该说的，然后就感觉特别不舒服。

R: 那你可以马上改口啊其实。

H: 对，但是我很怕这些话被拿出去乱传呀，而且有一些话我也不敢讲。你比如说，有一门课学生是要求读英文文献的。然后有次他们就问我，去哪里找这些文献，我就说了谷歌学术。然后就有学生问，是不是他们得翻墙去找，然后我就不敢再往下讲了。VPN在国内是明令禁止的。我刚回国，我不知道这个我能讲到哪种程度。

Excerpt 4: Participation rules in online linguistics classes

H: 课堂参与占期末总分的10%。每节课的分数分为三档，0分、7分和10分。0分就意味着你整节课都没有说话。只要你主动发言了，你就能得到7分。如果你的观点特别好，就是挺有思想深度的，你就能得到10分。最后期末的课堂参与总分不是每节课的平均分，是你拿到过的最高分。我的学生都是那种从小考试考到大的，他们就特别想要去刷分你知道么，就是那些还没有开口说过话的就拼命抓住机会说，那些已经拿到7分的就想继续在课上贡献更好的观点。

Excerpt 7: Adaptation to unexpected incidents in remote classes

H: 我现在就感觉对一些事情的容忍度提高了，就是没有那么容易就抓狂这样。如果学生说他们那边的网特别慢，我就说没关系你可以回头看课程的回放什么的。有的时候我那个麦就有点问题，然后学生就会特别淡定地提醒我说，他们听不到这样。又或者有的时候纯属是我讲课的问题。总之，我现在对很多事情都见怪不怪了。
